# Comparison of single and double autologous stem cell transplantation in multiple myeloma patients

**DOI:** 10.1515/med-2021-0216

**Published:** 2021-01-27

**Authors:** Umit Yavuz Malkan, Haluk Demiroglu, Yahya Buyukasik, Ayse Karatas, Elifcan Aladag, Hakan Goker

**Affiliations:** Department of Hematology, University of Health Sciences, Diskapi Yildirim Beyazit Training and Research Hospital, Ankara, Turkey; Department of Hematology, Faculty of Medicine, Hacettepe University, 06230, Ankara, Turkey

**Keywords:** second autologous stem cell transplantation, multiple myeloma

## Abstract

**Background:**

Autologous stem cell transplantation (ASCT) is one of the standard treatments of choice for eligible multiple myeloma (MM) patients. Herein, we aimed to analyze MM patients at our center and compare the clinical outcomes of single and double ASCT patients.

**Materials and methods:**

Patients who were diagnosed as having MM and had undergone single or double ASCT in our clinic between the years 2003 and 2020 were retrospectively examined.

**Results:**

In this study, the median time of second ASCT is approximately 3.6 years from the first ASCT. Overall survival (OS) duration of the single and double transplanted groups was 4,011 ± 266 vs 3,526 ± 326 days, respectively (*p*: 0.33). Progression-free survival (PFS) duration of the single and double transplanted groups was 2,344 ± 228 vs 685 ± 120 days, respectively (*p*: 0.22). Disease assessment after ASCT stable or progressive disease, partial remission, and very good partial or complete remission (CR) in single and double ASCT groups was 62/44/105 and 8/4/5, respectively (*p*: 0.22).

**Conclusion:**

The present study points out that the second ASCT treatment option for MM patients may not be effective as suggested, especially in the era of novel MM drugs, since our results come from the past data that novel drugs were not exist. In conclusion, we found no benefit with second ASCT in MM patients in terms of PFS and OS or CR rates, and the novel anti-myeloma drugs might decrease the need for a second transplant.

## 1 Introduction

Multiple myeloma (MM) is the second most common hematological malignancy, and autologous stem cell transplantation (ASCT) is the standard treatment of choice for eligible MM patients [1]. High-dose chemotherapy and ASCT have greatly changed the clinical course of MM [2,3]. Although high-dose therapy with ASCT is not curative, progression-free survival (PFS) and overall survival (OS) are prolonged compared to the standard-dose myeloma treatments alone. The median OS of MM patients is now ranging between 6 and 10 years with the help of novel agents [4].

Treatment choices for relapsed MM after an ASCT are a second ASCT, nonmyeloablative allogeneic hematopoietic cell transplant (HCT) as part of a clinical trial, and treatment with salvage chemotherapy. ASCT remains to be one of the main treatment options for MM. Many studies tried to find the best way of this procedure to maximize the benefit for the patients. There is a need for further studies regarding ASCT in MM patients. Herein, we aimed to analyze MM patients at our center and compare the clinical outcomes of single and double ASCT patients.

## 2 Patients and method

### 2.1 Study design and data collection

This study has been designed retrospectively. The patients who were diagnosed as having MM and had undergone ASCT in our hematology clinic between the years 2003 and 2020 were examined. Demographic data, transplantation data, and posttransplantation updates of the patients were obtained from the hospital database. Patients’ age, gender, dates of diagnosis–relapse–transplantation–exitus, M-protein types and levels, International Staging System (ISS) stage, Eastern Cooperative Oncology Group (ECOG) score, hemoglobin, sedimentation, calcium, uric acid, creatinine, albumin, β2-microglobulin, vitamin D levels, cytogenetic analyses, treatments, conditioning regimens, and disease status before ASCT and after treatment were noted.

All of the ethical considerations had been strictly followed by the Declaration of Helsinki 1964. As a standard care/action of the hospitals of the Hacettepe Medical School, it has been recognized from the patient records that all of the studied patients had given informed consent at the time of hospitalization and before the administration of chemotherapy and other relevant diagnostic/therapeutic standard of care.

### 2.2 Patients and disease characteristics

The patients who were aged ≥18 years, at the time of MM diagnosis, and underwent ASCT procedure were included in this study. Some of the patients had only one ASCT, whereas some other group of patients had double ASCT. The second ASCT was performed in eligible patients who had elevated serum or urine M-protein levels, elevated bone marrow atypical plasma cell ratio, the occurrence of new lytic bone lesions or plasmacytoma, and any other evidence of clinical progression or relapse. There were 17 patients in the double ASCT group and 211 patients in the single ASCT group. All of the patients who were double transplanted had relapsed before their second ASCT. All of the single or double ASCT patients were in ECOG 1 performance status before the procedure. There was no significant comorbidity from concomitant severe diseases in any patient. All patients underwent ASCT after receiving four to eight courses of induction chemotherapy. They received bortezomib/cyclophosphamide/dexamethasone, bortezomib/dexamethasone, vincristine, doxorubicin, and dexamethasone, and bortezomib/thalidomide/dexamethasone as induction therapy. Patients who underwent double ASCT also received proteasome inhibitors and immunomodulatory agents before the second ASCT. The preparative regimen for single or double ASCT patients was melphalan 200 mg/m^2^. The patients did not receive total body irradiation. There was no difference of preparation between the two groups. We did not give maintenance treatment after the transplantation procedure. No patient was given interferon. Responses were determined according to the International Myeloma Working Group response criteria. Cytogenetic data were available only for a minority of patients and were not considered in this analysis.

### 2.3 Statistical analysis

The primary endpoint of the study is the OS duration. OS was calculated from the start date of the first therapy to death for any cause. PFS duration was calculated from the transplantation date (second transplantation date was taken for double transplanted patients) to relapse or progression date. The patients who did not die and those who did not relapse or die in remission at the last follow-up were censored at this time for OS and PFS computations, respectively. Transplantation-related mortality (TRM) included any death occurring within 90 days and attributable to high-dose therapy.

The SPSS software version 25 (SPSS Inc., Chicago, IL, USA) was used for analyses. Continuous and categorical data were compared using the *t*-test and chi-square test, respectively. The variables were investigated using analytical methods (the Kolmogorov–Smirnov/Shapiro–Wilk’s tests) to determine whether they are normally distributed or not. One-way analysis of variance was used to compare parameters using means and standard deviations for normally distributed variables. The Kruskal–Wallis test was used to compare parameters for non-normally distributed variables. Survival analyses were done using the Kaplan–Meier test. Multivariate analysis of predictors of survival was performed using the Cox regression test. Parameters with *p* values ≤0.15 in univariate tests were included in the multivariate analysis. The *p* values <0.05 were considered statistically significant.

## 3 Results

**Figure 1:**
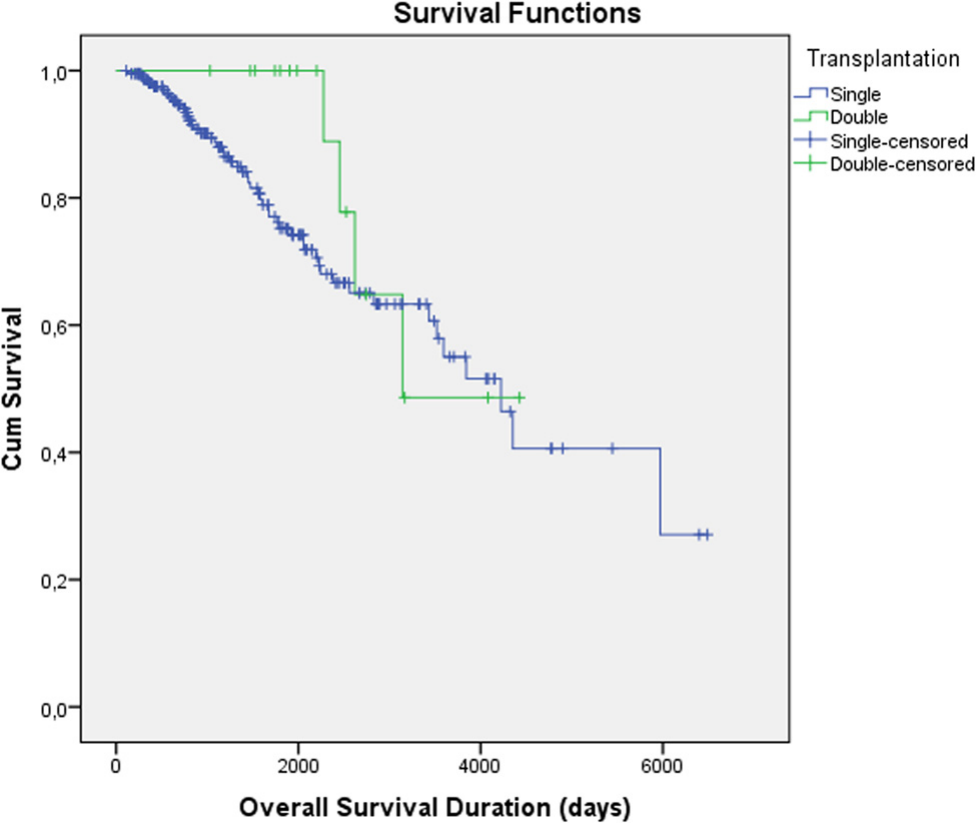
There is no statistically significant overall survival duration difference between the single and double transplanted groups (4,011 ± 266 vs 3,526 ± 326 days, respectively, *p*: 0.33).

**Figure 2:**
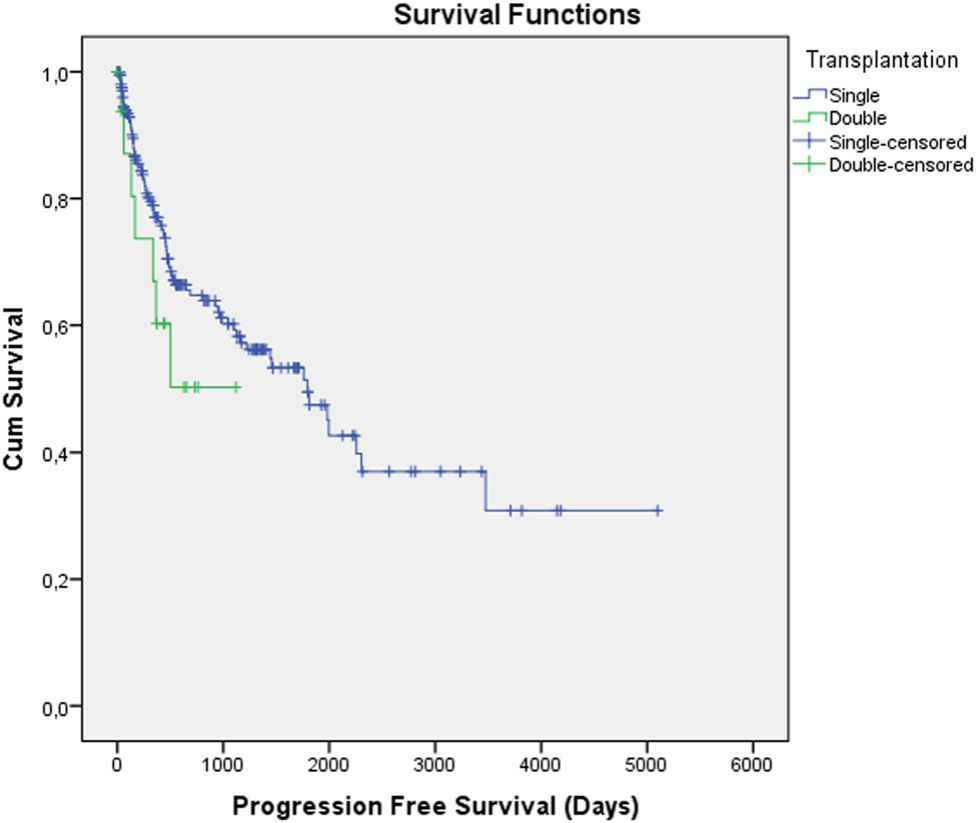
There is no statistically significant progression-free survival duration difference between the single and double transplanted groups (2,344 ± 228 vs 685 ± 120 days, respectively, *p*: 0.22).

The baseline characteristics of the patients in the two groups are similar except gender and serum β2-microglobulin level (Table 1). Disease assessment after ASCT stable or progressive disease, partial remission (PR), and very good partial or complete remission (CR) in single and double ASCT groups was 62/44/105 and 8/4/5, respectively (*p*: 0.22). Among the double transplanted patients, five were transplanted within one year after the first transplant. The median duration between the first and second transplants was 1,322 (414–4,242) days in double ASCT patients. OS duration of the single and double transplanted groups was 4,011 ± 266 vs 3,526 ± 326 days, respectively (*p*: 0.33). There was no statistically significant difference between the OS duration of single and double ASCT patients (Figure 1). Only four patients had died from TRM in the single ASCT group, whereas no patients had died from TRM in the double ASCT group. The PFS duration of the single and double transplanted groups was 2,344 ± 228 vs 685 ± 120 days, respectively (*p*: 0.22). There was no statistically significant difference between the PFS duration of single and double ASCT patients (Figure 2). The factors that are related to the OS of double ASCT patients were analyzed. In univariate analysis, serum calcium levels and IgA-type M-protein were found to be related to the OS of double ASCT patients. The mean OS time was 3,660 days [95% confidence interval; 2,997–4,323 days] in patients who had calcium levels <10 mg/dL, whereas it was 2,454 days [95% confidence interval; 2,152–2,786 days] in patients who had calcium levels of 10 g/dL or above (*p*: 0.09). The mean OS time was 1,856 days [95% confidence interval; 1,741–1,985 days] in patients who had IgA-type M-protein, whereas it was 2,729 days [95% confidence interval; 1,031–4,427 days] in patients who had IgG-type M-protein and 2,047 days [95% confidence interval; 1,473–2,621 days] in patients who had kappa/lambda light-chain M-protein (*p*: 0.06). However, this relationship was not found in multivariate analysis (*p* > 0.9 for serum calcium and IgA-type M-protein). In univariate analysis, serum uric acid levels and β2-microglobulin were found to be related to the PFS of double ASCT patients. The mean PFS time was 576 days [95% confidence interval; 34–1,118 days] in patients who had serum uric acid level <5 mg/dL, whereas it was 186 days [95% confidence interval; 35–371 days] in patients who had serum uric acid levels of 5 mg/dL or above (*p*: 0.04). The mean PFS time was 844 days [95% confidence interval; 533–1,156 days] in patients who had β2-microglobulin <3 mg/L, whereas it was 325 days [95% confidence interval; 148–501 days] in patients who had β2-microglobulin of 3 mg/dL or above (*p*: 0.07). However, this relationship was not found in multivariate analysis (*p* > 0.35 for uric acid and β2-microglobulin).

**Table 1:** The baseline characteristics of the patients in the single and double ASCT groups

Parameters	Single ASCT (N:211)	Double ASCT (N:17)	*p* value
Age (years)	55 (34–76)	54 (42–65)	0.56
Gender (F/M)	91/120	3/14	**0.04**
M-protein type (IgA/IgG/light-chain disease/IgD/IgE/nonsecretory)	39/114/50/4/1/3	3/9/5/0/0/0	0.97
Disease assessment before ASCT (SD or PD/PR/VGPR or CR)	20/122/69	4/7/6	0.16
Disease last assessment (SD or PD/PR/VGPR or CR)	62/44/105	8/4/5	0.22
Serum M-protein (mg/dL)	3,140 (26–72,700)	3,940 (1,000–17,100)	0.97
Exitus/alive	51/160	4/13	0.95
ISS (I/II/III)	80/52/47	11/6/0	0.05
Bone lesion at diagnosis (N/Y)	69/142	3/14	0.19
β2-microglobulin (mg/L)	4.91	2.88	**<0.001**
Serum vitamin D (ng/mL)	18.8 (5.0–78.4)	19.8 (9.1–39.0)	0.87
Serum hemoglobin (g/dL)	11.2 (3.0–16.6)	11.3 (6.1–14.2)	0.99
Sedimentation (mm/h)	55 (2–144)	53.5 (2–147)	0.88
Serum calcium (mg/dL)	9.5 (6.8–18.4)	9.6 (5.8–13.6)	0.82
Serum uric acid (mg/dL)	5.9 (2.3–17.2)	5.0 (2.1–14.6)	0.19
Serum creatinine (mg/dL)	0.98 (0.40–13.81)	0.92 (0.72–2.64)	0.31
Serum albumin (mg/dL)	3.9 (2.3–5.0)	3.9 (2.3–4.8)	0.78

The significance of bold value is *p* < 0.05.

Y: Yes; N: No; SD: stable disease; PD: progressive disease; PR: partial remission; VGPR: very good partial remission; CR: complete remission.

## 4 Discussion

Treatment options for relapsed MM after an ASCT include a second ASCT, nonmyeloablative allogeneic HCT as part of a clinical trial, and treatment with salvage chemotherapy. ASCT remains to be one of the main treatment options for MM. Many studies tried to find the best way of this procedure to maximize the benefit for the patients. In general, a second ASCT is not recommended for cases that relapse within 12–18 months (if no maintenance therapy was given) of the first, since the PFS following the second ASCT will most likely be even shorter than the benefit seen with the first transplant [5]. Similarly in the present study, the median duration between the first and second ASCT was 1,322 days in double ASCT patients. In the cases that were given lenalidomide maintenance, a second ASCT is not considered if the relapse occurs within 36 months of the first ASCT. These patients are best treated with active agents that they have not received before or have had good responses to in the past as well as clinical trials investigating novel therapies. Contrary to this literature knowledge, we did not give any maintenance treatment for our MM patients who were undergone ASCT.

For patients achieving complete or near-complete response with the first ASCT, it was suggested to reserve the cryopreserved stem cells for a second ASCT to be used at the time of relapse [6,7]. On the other hand, in our present study, we have performed ASCT to patients with stable or progressive disease, partial remission, and very good partial remission or CR.

In the literature, two randomized trials compared second ASCT vs treatment with chemotherapy alone in patients with late relapse after a first ASCT. After a median follow-up of 31 months, second ASCT resulted in a longer median time to progression (19 vs 11 months; hazard ratio 0.36) [8]. In another study, after a median follow-up of 37 months, PFS (median 21 vs 19 months) and OS (three-year OS, 72% each) were similar in the two treatment arms on intention-to-treat analysis [9]. In our present study, we did not find any benefit with double ASCT in MM patients in terms of PFS and OS or CR rates.

In another study, a single institution retrospectively analyzed 200 patients with MM who received a second ASCT after recurrence following initial therapy that included an ASCT (37% tandem) [10]. A partial or greater response was seen in 80% by Day 100. At a median follow-up of 57 months, the median PFS and OS times following the second ASCT were 15 and 42 months, respectively. Results were worse among cases that had an initial remission duration of <18 months and those who had less than a PR to re-induction therapy prior to ASCT.

The role of tandem ASCT as an upfront treatment in cases with MM and its advantage over single ASCT are a matter of debate [11,12]. The development of extramedullary plasmacytomas during proteasome inhibitor treatment is also associated with an unfavorable prognosis in MM [13], and tandem ASCT could be considered in such cases. Typically, in tandem autologous transplant, two autologous transplants are performed within a period of no more than 6 months. However, it was stated in the literature that if a second ASCT is contemplated, it is preferable to perform the procedure within 6–12 months of the first transplant [14]. In our study, the median time of second ASCT is approximately 3.6 years from the first ASCT indicating that our patients did not undergo tandem ASCT. In our study, the second ASCT was performed to patients who had elevations in serum or urine M-protein levels, elevation in bone marrow atypical plasma cells ratio, the occurrence of new lytic bone lesions or plasmacytoma, and any other evidence of clinical progression or relapse. Eventually, all of our patients had relapsed after the first ASCT. The relapse in our study represents the relapse of the disease detected by laboratory tests, imaging techniques, or bone marrow investigation.

Many studies have investigated the efficacy of ASCT in the relapse MM, and they have showed that ASCT for a second or even a third time is an efficient treatment choice for MM patients who had previously undergone ASCT procedure [15,16,17]. In a previous study, it was stated that one-year nonrelapse mortality of 2% and three-year OS of 46% were found in patients treated with a second ASCT. These data favor the second ASCT that is a safe and efficient treatment option for relapse MM [18]. Moreover, patients with a long relapse-free interval from previous ASCT (>36 months) had longer PFS (*p*: 0.045) and OS (*p*: 0.019) in comparison with patients with a shorter relapse-free interval (<36 months) [18]. In another study, second ASCT was found to be safe (TRM, 6%) and effective (CR rate: 44%; median PFS: 14 months) case that had been treated with maintenance agents after upfront ASCT [19]. In another study, 93% of the cases reached response after second ASCT, with 46% of them having a very good partial remission. The median PFS after second ASCT was 18 months, and no patients lost due to treatment [20]. The prospective studies are valuable for relapse MM patients investing the role of ASCT. In a study in which the patients were given a bortezomib-based re-induction after relapse MM, they were randomized to second ASCT or cyclophosphamide. Second ASCT prolonged the median PFS (19 vs 11 months; *p* < 0.001), but not OS (65 vs 56 months; *p*: 0.19), when compared to cyclophosphamide [21]. The comparison of the conventional treatment with second ASCT in relapse MM is another important question. In a study that compares second ASCT with conventional chemotherapy in cases that previously undergone ASCT, second ASCT prolonged the median OS (56 vs 25 months; *p*: 0.04) when compared to conventional chemotherapy [22]. All these data favor that the second ASCT is a safe and efficient treatment choice for the relapsed and refractory MM cases. On the other hand, the novel anti-myeloma drugs might change the need for a second transplant since these agents are much more effective than previous conventional myeloma therapies. The selected patients who had prolonged remission from first ASCT and adequate performance status might have the most beneficial group for second ASCT in the novel myeloma drug era.

The main limitations of our study are the relatively low number of patients and its retrospective design. On the other hand, our study included the analysis of previously treated patients between years 2003 and 2020; therefore, the treatment strategies for MM were changed during these 17 years. In the past years, novel MM treatment agents such as carfilzomib, ixazomib, daratumumab, lenalidomide, and other drugs were not available, and the treatment algorithm for MM was poorer and simpler than the present one.

## 5 Conclusion

To summarize, we found no benefit with second ASCT in MM patients in terms of PFS and OS or CR rates. There may be several reasons that lead to these results. First, the ISS stage of our double ASCT group was lower than that of single ASCT group. Second, the double ASCT patient group had a relatively low number of patients. The present study points out that the second ASCT treatment option in MM may not be effective as suggested, especially in the era of novel MM drugs, since our results come from the past data that novel drugs were not exist. The generalizability of the data presented here should be confirmed by future prospective studies. In conclusion, we found no benefit with second ASCT in MM patients in terms of PFS and OS or CR rates, and the novel anti-myeloma drugs might decrease the need for a second transplant.
